# Improved semi-supervised autoencoder for deception detection

**DOI:** 10.1371/journal.pone.0223361

**Published:** 2019-10-08

**Authors:** Hongliang Fu, Peizhi Lei, Huawei Tao, Li Zhao, Jing Yang

**Affiliations:** 1 School of Information Science and Engineering, Henan University of Technology, Zhengzhou, China; 2 Key Laboratory of Underwater Acoustic signal Processing of Ministry of Education, Southeast University, Nanjing, China; Newcastle University, UNITED KINGDOM

## Abstract

Existing algorithms of speech-based deception detection are severely restricted by the lack of sufficient number of labelled data. However, a large amount of easily available unlabelled data has not been utilized in reality. To solve this problem, this paper proposes a semi-supervised additive noise autoencoder model for deception detection. This model updates and optimizes the semi-supervised autoencoder and it consists of two layers of encoder and decoder, and a classifier. Firstly, it changes the activation function of the hidden layer in network according to the characteristics of the deception speech. Secondly, in order to prevent over-fitting during training, the specific ratio dropout is added at each layer cautiously. Finally, we directly connected the supervised classification task in the output of encoder to make the network more concise and efficient. Using the feature set specified by the INTERSPEECH 2009 Emotion Challenge, the experimental results on Columbia-SRI-Colorado (CSC) corpus and our own deception corpus show that the proposed model can achieve more advanced performance than other alternative methods with only a small amount of labelled data.

## Introduction

The importance of deception detection is self-evident. Deception refers to the act of covering up the truth with the false words, which not only causes the lack of trust between people, but also causes serious social harm. Therefore, people have been trying various methods to detect deception. At the end of the last century, people began to notice the difference between lie voice and normal voice. Ekman et al. collected the speech of lies and truths by investigating people about their perceptions on some films and television as the object of deception research, and found that the fundamental frequency of lies is obviously improved compared with normal speech [1]. The researchers at Purdue University detected deception by amplitude modulation and frequency modulation. The result shows that the features of Teager Energy-related have the possibility of distinguishing deception [2]. This shows that when people lie, their voice changes in pitch, pauses and etc. due to stress. Moreover, compared with the previous methods, the deception of detection based on speech can get rid of the constraints of time and space, making the results more objective [3]. Therefore, speech-based deception detection has important practical significance and great practical value.

However, the related research started late and researchers are studying from three aspects at present: speech feature extraction, feature processing and classification. The research team at Columbia University used composite features of speech to achieve good results with support vector machine (SVM) as a classifier [4]. The team of Professor Heming Zhao from Soochow University extracted the non-liner dynamic and prosodic features of speech, and used the relevance vector machine to detect deception [5]. At the same time, people hope to get more valuable features to help classification, so how to deal with speech features effectively has attracted people’s attention. In 2006, Hinton and Salakhutdinov initialized the multi-layer feedforward neural network Boltzmann machine (RBM) by priori information gained from unsupervised learning [6]. Later, a variety of unsupervised learning models were proposed, such as Deep Boltzmann Machines (DBM) [7], Autoencoder (AE) [8], Deep Belief Network (DBN) [9]. A DBN consists of a stack of Restricted Boltzmann Machines (RBMs), a DBM is a RBM with multiple hidden layers. The difference between DBN, DBM and Autoencoder (AE) is that AE find the characteristic representation of input data through non-linear transformation, which is a deterministic model; while DBN and DBM train around probability distribution, which extracts high-level representation through probability distribution (energy function) of input data, both DBN and DBM belong to a probabilistic model. DBN and DBM advocate greedy layer-wise pre-training using the unlabelled data, followed by a “fine-tuning” step using the labelled data. The core of this idea is that the high-level representations can be built from a large supply of unlabelled data inputs and labelled data can then be used to only slightly fine-tune the model for a classification task, this is equivalent to conducting unsupervised learning and supervised learning in sequence. Some researchers have followed this line of thinking. Researchers at Rungta College of Engineering and Technology in India extracted features of energy, zero-crossing rate, etc. in deception speech and constructed a classification model which consist of artificial neural network and SVM [10]. Zhou Yan from Soochow University used depth features of speech obtained by deep confidence network and achieved good results detected by SVM [11]. Even if this approach has some advantages, unfortunately, it is risky to combine the unsupervised learning with supervised learning in this way. Because unsupervised learning does not know what information will be useful for classification task, it just aims to retain all the information to perfectly reconstruct the input data, while supervised learning only preserves important information that is useful for classification and drops redundant information. In this case, there is a potential conflict of interest between them, which will lead to a decline in generalization of the model and affect classification performance. This conflict between supervised learning and unsupervised learning is also documented in reference [12, 13, 14], and the authors of these articles attempt to conduct them simultaneously to solve this problem. This paper will also refer to this idea for research, the details will be introduced below. Moreover, there is another problem in the field of speech-based deception detection, that is, it is very difficult to obtain a large amount of labelled data because tagging the data is very cumbersome and a lot of manpower and material resources is required in this course. More critically, there is no uniform marking criterion so that different people may give different labels for the same speech. In contrast, obtaining a good deal of unlabelled speech data from the internet is fairly inexpensive and plenty. Therefore, this paper mainly focuses on how to utilize labelled data and unlabelled data more efficient, that is, applies semi-supervised learning to data to achieve better deception detection work.

In other fields, some scholars have proposed a deep semi-supervised learning model in order to utilize unlabelled data [15]. For example, semi-supervised ladder networks and variational autoencoders [13, 16] were introduced, which achieved satisfactory accuracy with only a few hundred labels in image classification. Recently, Deng et al proposed semi-supervised autoencoder (SS-AE) for speech emotion recognition [14], which not only combines the autoencoder and classifier, but also categorizes unlabelled data into an additional class, excellent performance of this model can be showed by the results on the different data sets. Inspired by this, we tried to apply his semi-supervised autoencoder to speech-based deception detection but the results are not satisfactory. The reason for this is that the special change of people’s pronunciation when they lie lead to the feature distribution of deception speech is different from other phonetic features, and the deception detection only needs to determine whether it is a lie. Therefore, when we combine deception detection and semi-supervised learning, we need to adapt the model to the characteristics of deception speech. Based on this, we propose semi-supervised additive noise autoencoder (SS-ANE) for deception detection, which improves the existing semi-supervised autoencoder from the aspects of activation function and network structure. It not only reduces the dependence on a large number of labelled data, but also integrates information from labelled and unlabelled data to facilitate classification. To the best of our knowledge, this is the first work that deep semi-supervised model is used for speech-based deception detection.

Our contribution can be summarized as follows:

- A full Chinese speech data is constructed which containing carefully selected truths and lies. The corpus is the basis for speech-based deception detection, despite some researchers in the United States and Switzerland have established a deception corpus, but there is almost not an all-Chinese corpus. This paper builds a Chinese-based deception corpus in the way of other researchers to successfully establish a deception database, and supplements the speech data for the existing deception detection work.
- Different from the existing method of autoencoder-based deception detection, our proposed model is more adaptable to the deception detection work, and extends the application scope of the autoencoder in unsupervised learning to the supervised classification, so that it can extract data features and realize data classification simultaneously.
- Most methods of speech-based deception detection are limited to sufficient labelled speech data. In this paper, we show for the first time the semi-supervised autoencoder applied to deception detection. The experimental results on the CSC library and the self-built lie library show that our model can get impressed accuracy with a small number of labelled data.

## Methodology

### Semi-supervised autoencoder

**Fig 1:**
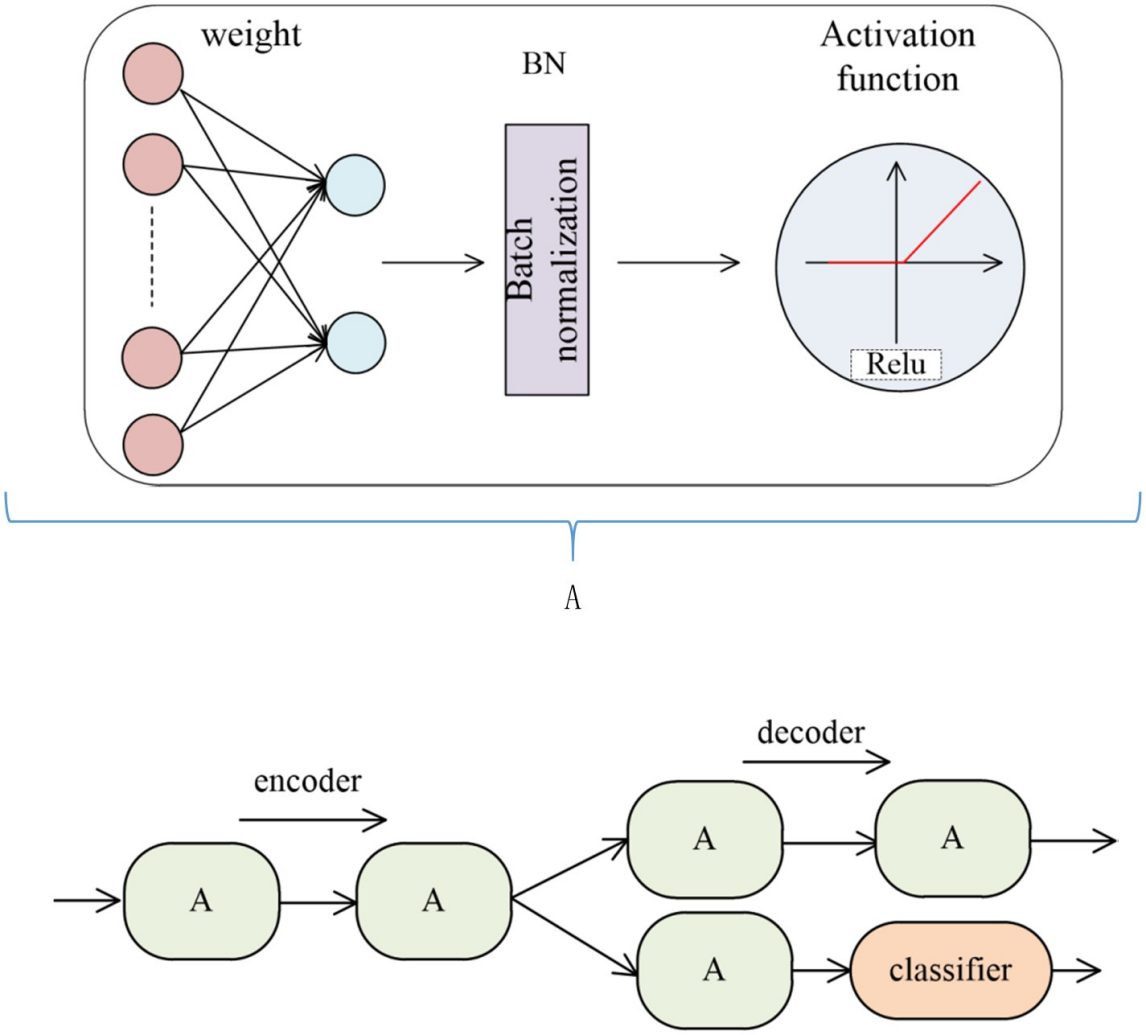
Semi-supervised autoencoder model.

In this part we introduce the Semi-supervised autoencoder (SS-AE) which proposed by Deng et al [14]. In paper 14, SS-AE is a multi-layer neural network which integrates supervised learning and unsupervised learning and each parts are composed of several hidden layers A in series. The parts contained in Hidden Layer A and the whole model framework are shown in Fig 1. Encoder is used to extract high-order features (that is, low-dimensional vectors) of original data, then the decoder in the first half maps the features to the output to reconstruct the data. The purpose is to make the coded features have all the information of the original data as far as possible. The lower half classifies the low-dimensional vectors after several layers of neural networks, so as to ensure that the coded features have classification information.

Basically, when both the coding network and the decoding network have only one layer, the coding part can be expressed as:

$$Y=S(W_{1}*X+B_{1})$$ (1)

For the decoding part:

$$Z=S(W_{2}*Y+B_{2})$$ (2)

Among them, S is a non-linear activation function, generally sigmoi or Relu, W1, W2 is the weight matrix, B1, B2 is the bias vector, Y is the encoded data, and Z is the output of the decoder. This model is essentially a variant of autoencoder, so we can add some constraints to make it produce interesting changes and adapt to different work, such as adding random disturbed signal to input data, so that the encoder can extract more robust features, or adding sparse constraints to the network, then the encoder can extract sparse features and so on.

### Semi-supervised additive noise autoencoder

#### Algorithm process

**Fig 2:**
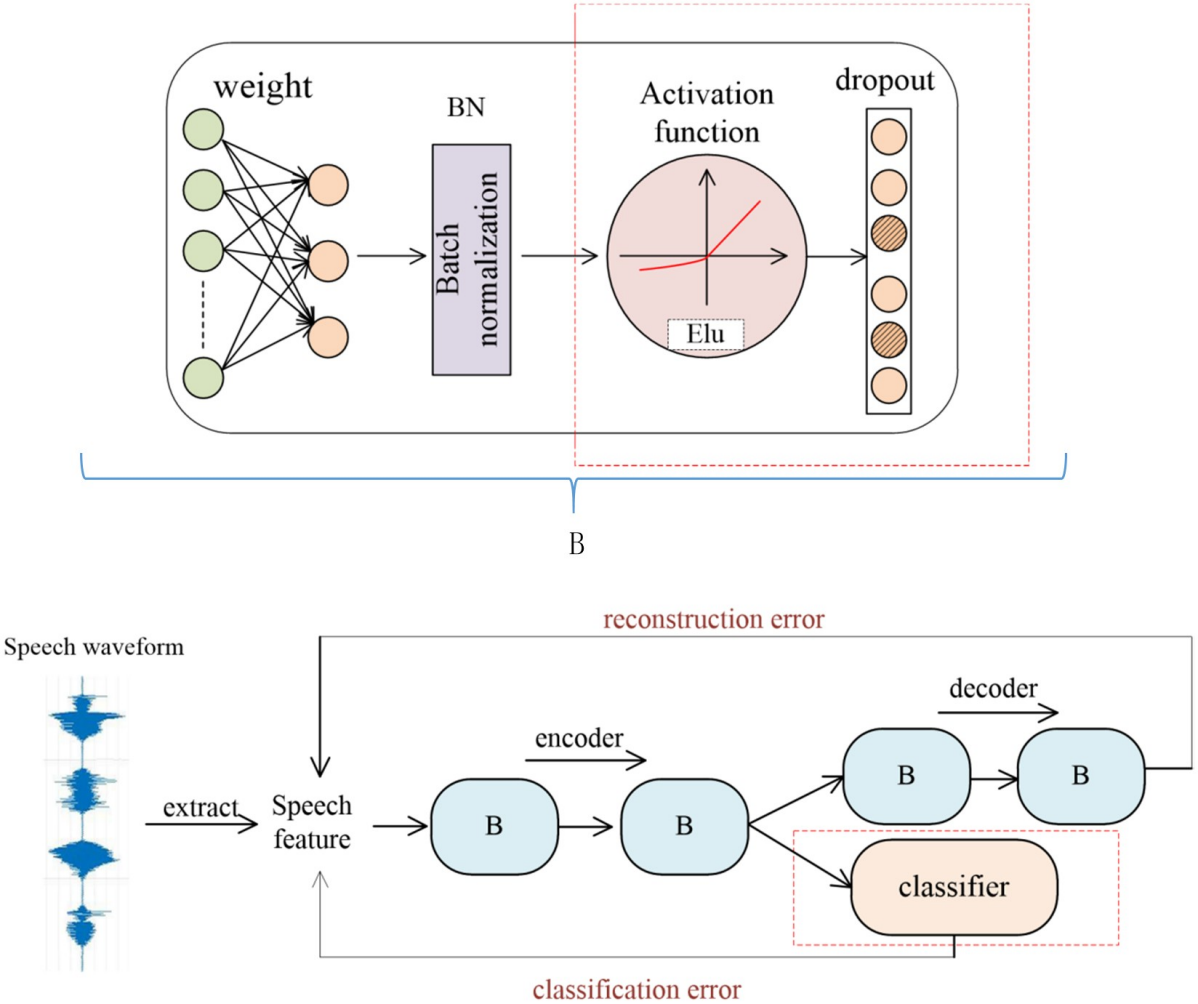
Structure of semi-supervised additive noise autoencoder.

SS-AE performs well in speech emotion recognition, but deception detection and emotion recognition belong to different classification tasks. Therefore, we have improved the SS-AE according to the characteristics of deception speech, so that it can more suitable for the discrimination of lies. As shown in Fig 2, Inside the red wireframe is the part of our improvement on the SS-AE proposed in paper 14. According to the characteristics of deceptive speech, we replace the original activation function with Elu and then add dropout to each layer of the network. Besides, the classifier is directly related to the encoder. The whole model framework is also shown in Fig 2. The encoding and decoding network are all connected by several hidden layers B. Each hidden layer B contains weights, batch normalization and activation functions, dropout.

Suppose we have a corpus with N labelled data {(x_1_, y_1_), (x_2_, y_2_)….(x_N_, y_N_))} and M unlabeled data {x_N+1_, x_N+2_….x_N+M_}, where y is the label corresponding to the data with K classes. Our purpose is to learn the distribution P(y|x) from these data. Following is an introduction of the complete calculation process after the data input model.

(1) For unsupervised learning, it consists of a deep additive noise autoencoder, which has multiple hidden layers to extract the depth features of the data. The input data is converted into a new expression through a multi-layer feedforward neural network nonlinearly in coding part, given an input x, the process is as follows:

$$\varepsilon_{e}^{1}=x$$ (3)

$$\varepsilon_{e}^{l}=Elu(w_{e}^{l-1}*\varepsilon_{e}^{l-1}+b_{e}^{l-1}),2\leq l\leq L$$ (4)

$$y_{e}^{L}=\varepsilon_{e}^{L}$$ (5)

The decoding part reconstructs the encoded data into the same data as the input data dimension:

$$\varepsilon_{d}^{1}=y_{e}^{L}$$ (6)

$$\varepsilon_{d}^{l}=Elu(w_{d}^{l-1}*\varepsilon_{d}^{l-1}+b_{d}^{l-1}),2\leq l\leq L-1$$ (7)

$$\varepsilon_{d}^{L}=w_{d}^{L-1}*\varepsilon_{d}^{L-1}+b_{d}^{L}$$ (8)

$$\bar{x}=\varepsilon_{d}^{L}$$ (9)

Where L represents the number of layers of the network, w and b are the inter-layer weights and bias respectively, which are parameters that need to be optimized. After the above steps, the loss function of the unsupervised learning part, that is, the reconstruction error of the data can be showed as:

$$C_{u}=\frac{1}{2}{\sum \left|x-\bar{x}\right|}^{2}$$ (10)

For supervised learning, We add an additional supervised classification task to the additive noise antoencoder. After the data is extracted from the encoding network, besides decoding, it is also input into the classifier for classification, and the process can be expressed as:

$$p=f(\beta *y_{e}^{L}+b)$$ (11)

Where p is the result of the classifier prediction, *β* is the inter-layer weight of the connected coding network and the classifier, and b is the bias. After this process, the classification error of the data, commonly known as cross entropy loss, can be computed as follows:

$$C_{s}=-\sum \text{log}(\frac{\text{exp}(p[y])}{\sum_{j=1}^{K}\text{exp}(p[j])})$$ (12)

y is the real label corresponding to the speech data, 1≤j≤K, K is the total number of categories of speech data which has been introduced in the previous paper. At this point, the forward propagation process of the model has been introduced.

(2) After the data passes through the network and the error is calculated, we use the gradient descent method to minimize the error and optimize the parameters. For unlabelled data, only data reconstruction is performed because there is no corresponding label, that is, the error function *C*_*u*_ is minimized. When training, the gradient of error function for each parameter is calculated first: $\left\{dw=\frac{\partial C}{\partial w},db=\frac{\partial C}{\partial b}\right\}$, then the parameters are optimized according to the gradient until the model converges. For the output layer, the error is:

$$\delta_{o}=\bar{x}*(1-\bar{x})*(\bar{x}-x)$$ (13)

For the upper layer of reconstructed output, the residual error of this layer is the value of output layer error after back propagation through weight:

$$\delta_{o-1}=\varepsilon^{L-1}*(1-\varepsilon^{L-1})*w^{L-1}*\delta_{L}$$ (14)

so the updated values of the layer parameters, that is, the gradient of this layer parameter and the updated parameter are:

$$dw=\varepsilon^{L-1}*\delta_{L},\;db=\delta_{L}$$ (15)

$$w^{L-1}=w^{L-1}-\lambda *dw,\;b^{L-1}=b^{L-1}-\lambda *db\;\left(\text{λ}\;\text{is}\;\text{the}\;\text{set}\;\text{learning}\;\text{rate}\right)$$ (16)

The other layers are deduced by analogy. According to the model sequence, when the residual error is back-propagated, it passes through the decoder and then passes through the encoder. Finally, all the parameters are updated and the first optimization is completed.

For labelled data, both reconstruction and classification are required, so the error function that needs to be minimized is:

$$C=C_{s}+\alpha C_{u}$$ (17)

*α* is the balance parameter. We also use the gradient descent method to iterate, different from when input is labelled data, we need to minimize the joint error function, so when the residual error of back propagation is going to enter the encoder, it is necessary to append the error of classification output layer additionally, the residual error of the classification output layer is:

$$\delta =\sum_{i=1}^{K}\left\{\frac{\text{exp}(y_{i})}{\sum_{j=1}^{K}\text{exp}(p[j])}-y_{i}\right\}$$ (18)

After that, the gradient calculation and parameter update method are the same as described above. In the training process, it is necessary to input the labelled data and the unlabeled data into the model at the same time, thus parameters will be optimized by minimizing the respective error function. A large amount of unlabelled data guarantees the generalization ability of the original feature learning, and the labelled data can make the learned feature more suitable for classification. Therefore, our model leverages the value of both labelled and unlabelled data.

#### Analysis of algorithm

**Fig 3:**
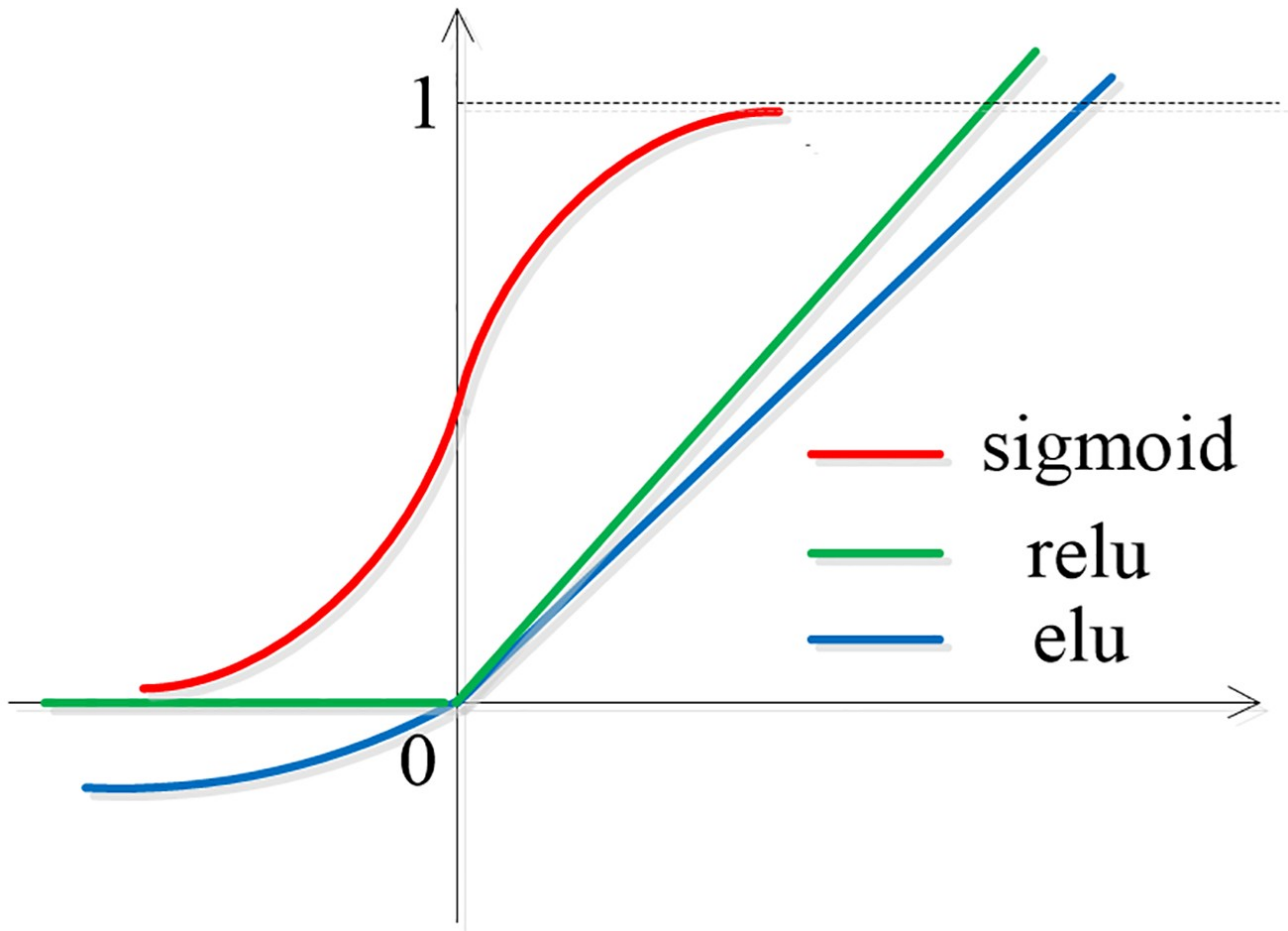
Diagram of activation function.

The role of the activation function in the neural network is very important. It realizes the non-linear transformation of data and makes the whole network more powerful in fitting data. Unlike previous autoencoders, we chose Elu as the activation function. As shown in Fig 3, if traditional activation function is applied for model such as Relu or sigmoid, when the input is negative or large negative values, the output of hidden nodes is 0, which is equivalent to the pseudo-dead state of units. When the model is optimized, the weights between these nodes and other nodes will change slowly or even produce gradient diffusion so that the information in these negative values will not be utilized validly and the terrible result is a decline in classification performance. In view of this, we choice Elu, as we can see that even if the input is negative value, the hidden nodes still generates response and the function changes smoothly in the part of the transverse axis less than 0, which ensures that the information in the negative value will not be wasted. In addition, the output mean of Elu is close to 0, which will make the network converge faster. We extract and standardize the features of CSC corpus and our own corpus, and the statistics display that the percentage of negative values in features is 24% in CSC corpus, and 28% in our own corpus. Therefore, it is more appropriate to select Elu as the activation function than the other two. Furthermore, different from the multi-classification of speech emotion recognition, deception detection based on speech only needs to judge whether the speech is lie, so that it can produce over-fitting when the number of training sample is small. Therefore, we prudently add a certain percentage of dropout to every layer of SS-AE to suspend the work of some hidden layer units with a certain probability, so as to prevent over-fitting. This change is also important for improving the accuracy rate. In addition, we use batch normalization to accelerate training in the network.

It is also a creative improvement for our model to train classifier by using the deep features extracted from the data after encoder directly. Data is transformed without additional neural networks, which ensures that the deep features obtained by encoder not only contribute to data reconstruction but also to classification. In addition, the direct connection of feature and classifier also simplifies the network structure, saves the calculation cost and speeds up the training.

**Fig 4:**
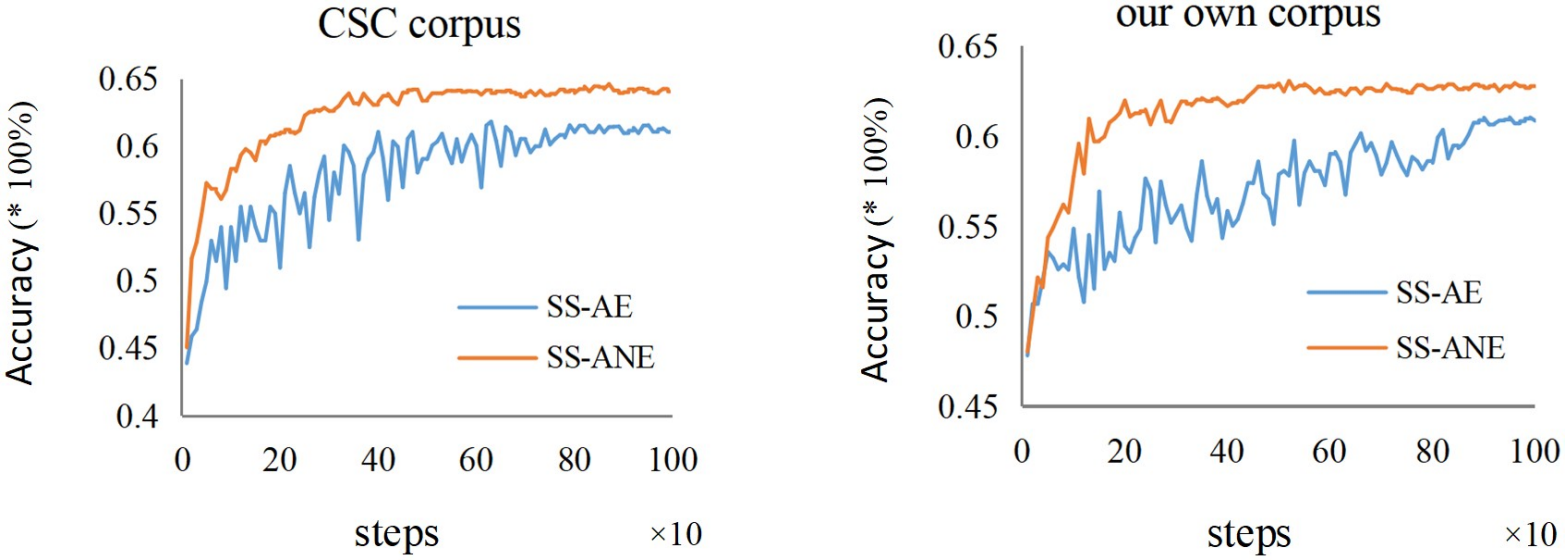
Accuracy rate of SS-ANE and SS-AE on the test set in two corpuses.

After the above steps, the improved model SS-ANE and the semi-supervised autoencoder (SS-AE) which proposed by Deng et al. were verified on the test set of two corpuses. We select 1000 labelled data in the CSC corpus and 200 labelled data in our own corpus (Detailed introduction of data selection can be found in the experiment part of this paper). It can be seen in Fig 4 that our model can achieve convergence faster and reach higher accuracy.

## Experiment

### Corpus and acoustic features

The premise of speech-based deception detection is a high quality corpus containing honesty and deception. The CSC corpus is a professional database designed for deception detection [17]. Students and teachers from Columbia University were invited to record this corpus, half of which were male. Subjects were told to participate in an activity called “Finding the Talents of the Top American Entrepreneurs”. Through communicating with the examiner, the subject needed to convince the examiner that he or she met the selection and eventually generated a speech sample of approximately 7.5 hours. 5411 valid speech segments were cut out for experimentation which include 2209 lies. Each speech lasted about 2 seconds. Finally, the training and test sets have 4328 and 1083 chunks.

In order to supplement the existing speech deception corpus and verify the effectiveness of our model, we have established a Chinese-wide deception corpus according to the rules earmarked by Tommy et al. to build Idiap Wolf Corpus [18], named “Killer”. We selected 50 hours of ultra-clear video of werewolf game and killer game. Game data can be downloaded from the following website: https://space.bilibili.com/250684483/channel/detail?cid=37791. These materials are all public and free videos from the Internet, and a manner which we accessed them conforms to the website’s terms and Chinese law. The number of players in each werewolf game is 12 and in each killer game is 16. Because of the game mechanism, some players will participate in multiple games repeatedly. Detailed number information after eliminating the number of repeats people is shown in Table 1. Then we used CoolEdit software to extract speech from video, removed the low-quality and hard-to-hear parts, moreover, different cutters were invited to auditory verification. In the end, 987 valid speeches were obtained including 468 lies. We have published all the extracted features and labels of these speeches, together with some speech texts, to the https://figshare.com/articles/features_and_label/9211553. Readers can download and experiment for free. Like CSC corpus, each speech lasted about 2 seconds. Among our corpus, 789 speeches were used as training sets and 198 speeches were used as test sets.

**Table 1:** Number of people participating in each game.

Game	male	female	total
Werewolf game	23	16	39
Killing game	40	24	64

The speech feature is the key to speech-based deception detection. The standard feature set of INTERSPEECH 2009 Emotion Challenge was selected for our experiment as shown in Table 2. In detail, there are 2*16 low-level descriptors which consist of zero crossing-rate (ZCR), harmonics-to—noise (HNR), Mel—frequency cepstral coefficient (MFCC) 1–12, root mean square (RMS) and 12 description functions with mean, maximum and minimum, mean square error, etc. This feature set contains the most widely used features and functions of acoustic features, each feature vector owns 2*16*12 = 384 dimensions [19]. To ensure the reproducibility of the experiment, the open source Opensmile toolkit was uesd to extract these features.

**Table 2:** LLDs and functionals.

LLDs(16*2)	Functionals(12)
(Δ)RMS Energy	standard deviation
(Δ)F0	kurtosis, skewness
(Δ)MFCC 1–12	linear regression: offset, slope, MSE
(Δ)ZCR	mean
(Δ)HNR	extremes: value, rel, position, range

### Experimental setup and evaluation metrics

First, Gaussian noise with a coefficient of 0.3 was added to the speech as the corrupted input data of the model. The coding and decoding network parts are both two layers, and each layer of the network sets the same number of hidden units. For the learning rate and the number of hidden units, we selected from {0.1, 0.01, 0.001}, {60, 120, 180} according to different experiments. The parameter *α* in the joint error function is chosen to be 1. During the training process, a small batch gradient descent method is used and a maximum of 1000 iterations are carried out to optimize the parameters. In addition, all data are standardized.

Accuracy rate was applied to evaluate performance of our model, which is the most commonly used evaluation indicator in the field of speech-based deception detection. Ten trials were performed for each model and take the average of the accuracy rate obtained from these ten times as the final result.

### Experimental results

We pay special attention to performance of proposed model with 500, 1000 labelled examples in CSC corpus and 100, 200 labelled examples in Killer corpus. Labelled data are randomly selected from the training set of each speech corpus, and the remaining data in the training set is treated as unlabelled data. The number of labelled data selected twice accounted for 10% and 20% of the two corpus respectively. Tables 3 and 4 shows the accuracy rate obtained by proposed model SS-ANE, SS-AE and other models which frequently used in the domain of speech-based deception detection. These models include: Support Vector Machine (SVM), which uses a linear kernel function with a C value of 1, Deep Neural Networks(DNN) [20] with three hidden layers each containing 256 units, StackedAutoencoder (SDAE) [21]+SVM, Deep Boltzmann Machines (DBM) [7]with three hidden layers(500,500,1000 hidden units), Deep Belief Network (DBN) [9], the DBN has three hidden layers and each hidden layer contains 1024 units.

**Table 3:** Average accuracy rate on the CSC test set with 500,1000 labelled examples.

model	labelled examples		
	500	1000	All
SVM	56.04%	58.57%	59.40%
DNN[20]	56.87%	59.46%	60.48%
SDAE+SVM[21]	57.75%	60.58%	61.63%
SS-AE[14]	58.01%	60.89%	
DBM[7]	57.61%	60.75%	61.86%
DBN[9]	58.44%	61.03%	62.88%
SS-ANE	59.52%	62.78%	

**Table 4:** Average accuracy rate on the Killer test set with 100,200 labelled examples.

model	labelled examples		
	100	200	All
SVM	57.82%	59.48%	60.04%
DNN[20]	58.34%	60.35%	61.08%
SDAE+SVM[21]	59.96%	61.10%	62.13%
SS-AE[14]	60.09%	61.48%	
DBM[7]	58.83%	60.59%	61.40%
DBN[9]	60.09%	61.62%	63.64%
SS-ANE	61.81%	63.89%	

As can be seen from the experimental results, our model can achieve the most advanced performance compared with other models when the same number of labelled data provided. our model obtains 59.52% and 62.78% accuracy rate with only 500 and 1000 labelled data one the CSC corpus, which is comparable with the best accuracy rate 62.88% obtained by DBN that uses all labelled data for training. And we report an accuracy rate of 61.81% and 63.89% on the Killer corpus with 100 and 200 labelled examples respectively, it is worth mentioning that our model still performs better even if other models use all data of training set as labelled data. These results show that our model can indeed reduce the dependence on labelled data. Besides, DBN and DBM also combine unsupervised learning with supervised learning, but the effect is not as good as our proposed model. As mentioned in the introduction, the purpose of unsupervised learning and supervised learning is different. DBN and DBM adopt the method of first unsupervised learning and then using supervised learning to fine-tune, so that a potential conflict of interest between them affects classification, which further proves the effectiveness of our method of conducting supervised learning and unsupervised learning simultaneously. In our semi-supervised autoencoder network, we used a additive noise autoencoder. In order to verify the influence of different autoencoder on the semi-supervised autoencoder network, Sparse autoencoder (SAE), Contractive autoencoder (CAE) these two classic autoencoders were selected as the experimental object, and experimental results as shown in the Tables 5 and 6. It can be seen that choosing different autoencoders will affect the performance of semi-supervised network. What is more, the additive noise autoencoder we chosen outperforms other autoencoders, which further proves the effectiveness of our model.

**Table 5:** On the CSC test set, results of the variant of autoencoders on our model.

autoencoder	labelled examples	
	500	1000
SAE	58.34%	60.48%
CAE	58.13%	59.97%
DAE	59.52%	62.78%

**Table 6:** On the Killer test set, results of the variant of autoencoders on our model.

autoencoder	labelled examples	
	100	200
SAE	59.60%	61.12%
CAE	58.16%	60.04%
DAE	61.81%	63.89%

## Conclusion and outlook

In view of the difficulty in obtaining speech label and a large amount of unlabelled data, we have mainly researched the application of semi-supervised learning in speech-based deception detection. Inspired by the existing deep semi-supervised model, we propose a semi-supervised additive noise autoencoder. The difference between this model and the traditional unsupervised autoencoder is that the depth speech features learned are both helpful for reconstruction and classification. The experimental results demonstrate that the proposed model can achieve advanced performance with only a small number of labelled data. This proves that our model can take advantage of the information in both labelled and unlabelled data and use it for speech-based deception detection.

In future research, we will further increase the number of network layers to extract deeper features, try to combine other models and autoencoder networks to achieve better performance on semi-supervised learning.
